# The Nursing Supplement

**Published:** 1890-03-01

**Authors:** 


Being the Extra Nursing Supplement of "The Hospital" Newspaper.

All Contributions for this Supplement should be addressed to the Editor, The Hospital, 140, Strand, London, W.C., and should have the word "Nursing" plainly written in left-hand top corner of the envelope.

### En Passant.

SHORT ITEMS.—The committee of Torbay Hospital propose to affiliate themselves to the Pension Fund by an annual payment of £20.—Burnley Victoria Hospital is going to increase the staff of the Nursing Institute.—The new matron of Salisbury has reorganised the nursing system, and is advertising for lady probationers.—Sister Rose Gertrude has permitted herself to be fêted at New York: this rose, it seems, was, after all, not born to blush unseen.—Miss Olga Hertz has written an account of the district nursing of Manchester for the *City News*.—A home for nurses is wanted at Hulme.—During the late row at Banstead Asylum one hundred nurses went to the house of one of the medical officers and cheered him.

EAST LONDON NURSING SOCIETY.—The total number of persons nursed in 1889 was 3,645, and 82,389 visits were recorded—against 3,021 nursed and 73,535 visited in the previous year. During the year two new districts have been opened—St. Mary's, Johnson Street, and St. Peter's, Hoxton Square. A new matron has been added to the staff, and there are now 25 nurses at work. During the past year several changes have taken place. Six nurses have resigned, one of whom, Nurse Gibbs, had worked for many years in St. Peter's, London Docks, and is now in receipt of a pension, mainly provided by that district, in recognition of her services. Another old servant, Nurse Clair, has been transferred to the post of maternity nurse.

NORTH LONDON NURSING ASSOCIATION.—This association nursed 2,421 cases last year, at a cost of £1,113. The visits of the nurses averaged 2,472 per month. The results were—739 recovered or convalescent, 94 transferred to hospitals, 234 deaths, 271 removed from books for other cases, and 100 were carried forward to 1890. The cost of nursing had been 15s. 8½d. per case; the cost per visit 9d., which was lower than any former record. The nursing staff maintained during the year consisted of nine nurses and superintendent. An anonymous donor who had sent on a former occasion £50 to the association sent a like sum, with instructions to divide it among the nurses; who, after accepting £10 for the purpose of purchasing sundry nursing requisites for the poor patients, unanimously agreed to hand over the balance to the treasurer, accompanied by a letter expressing their thankfulness in thus being able to render help.

THOUSANDS OF POUNDS FOR THE NATIONAL PENSION FUND.—We have the pleasure to acknowledge the following sums towards the £14,000 now being raised to at once bring the Donation Bonus Fund up to £40,000. We hope to be able to acknowledge contributions amounting to £1,000 every week until the £40,000 are completed. Contributions of any amount may be sent to the Editor of The Hospital, or to the Manager, National Pension Fund for Nurses, 8, King Street, Cheapside, London, E.C.:

The Sixth Thousand.

Amount already received **Six Thousand Pounds.**

THE LEEDS INSTITUTION.—Mr. Frederick Baines lately presided at the annual meeting of subscribers to this institution. The number of cases nursed last year was 762, and 228 applications had to be refused. The staff consisted of 75 nurses engaged in private nursing, 7 in district nursing, and 22 probationers in training at hospitals. Fourteen nurses had left the institution during the year, five out of this number to be married. One nurse had been dismissed for unsatisfactory conduct. Twelve had completed their training during the year. One probationer left the hospital where she was being trained at the end of three months, finding the work uncongenial; she paid the expenses incurred for that period. There had been frequent cases of illness amongst the nurses during the year. One contracted typhoid fever whilst attending a patient, necessitating her being off duty for five months. The sad death of Nurse Jessie Paine was referred to. Miss Dawson was heartily thanked for the successful way in which she manages the institution.

NAVAL NURSING SISTERS.—"A Pensioner" who has written a diatribe against Naval Nursing Sisters to the *Naval and Military Argus* is likely to get into trouble. A Naval Sister holds rank under the surgeon, she is always a lady by birth, and is ever an important person in her way. The letter is in very bad taste, and evidently prompted by personal malice, so that we cannot but wonder how our contemporary came to print it. That Naval Sisters do not have the hard ward work to do which hospital nurses have is true; but then before they can become Sisters they have all this hard training to go through. We quote a few lines of the letter merely to show its tone: "What are they? What do they do? Are they lady nurses, nursing sisters, or a medium between a sickbayman and the surgeon? Whatever they are, they are utterly useless in Naval hospitals; why should they not do their duty independent of the S.B.S.? Instead of their showing some gratitude towards the S.B.S. for the blunders they rectify for them, they do their utmost to get some trifling report against them. I hope I shall not have the misfortune of being a patient in a Naval hospital while these so-called Sisters are there."

SOUTH DEVON HOSPITAL.—The report of last year's work at this Plymouth hospital says that the work of the Nursing Institution was of exceptional interest and value, and in the scarlet fever epidemic the staff were at the call of the sick, not only in private families, but also in the Infectious Diseases Hospital of the Town Council. The eagerness with which the nurses had sought this special duty, and the cheerful spirit in which their services had been rendered, were especially noteworthy. Most satisfactory reports on their work had been received from the medical officer of health and from private families, and the committee felt that to Miss Hopkins the charity was largely indebted for the efficient training of the nurses. The earnings of the nurses amounted to £897, and there was after payment of expenses a balance left in hand of £318, with which it was proposed to form the nucleus of a fund for providing additional accommodation for the nurses. The committee had made arrangements for a nurse whose services should always be available for gratuitous nursing among the poor. The public are warned against nurses who represent themselves as trained at this hospital and so get appointments. Every nurse holding the certificate of the South Devon and East Cornwall Hospital is bound to have her certificate endorsed every six months by the matron.

### Nursing Lectures.

By Percy Lewis, M.D.

### Lecture IV.—THE CARE OF CHEST CASES.

With regard to covering in bed, chill is the chief thing to be avoided. It is a physiological fact that cold applied to the surface of the body produces congestion of the internal organs by driving the blood from the surface into them. Now congestion is the first stage of inflammation, and although in a healthy person a slight temporary congestion is unimportant, in one whose internal organs are already inflamed its effect can hardly be over-estimated. A slight chill in a person suffering from bronchitis or pneumonia may just be the determining factor as to whether a patient shall live or die. Flannel should be worn somehow, either as a vest inside, or flannel jacket outside. If the former, it should be changed at least every three days, and where possible the patient should have one for night and one for day. The bed clothes should be in sufficient quantity to keep him warm without being excessively hot. Avoid making them so heavy that they press on the chest and embarrass the breathing.

How to make poultices you will learn practically in the wards; still there are a few special points I may mention here. Avoid chill in applying and removing them. With this object, some physicians are in the habit of ordering two layers of flannel to be placed between the skin and the poultice, one layer only being removed with the latter. By this means not only is the patient protected from chill but also from the unpleasant sensation of having the poultice next his skin, a point specially useful in children. A poultice must not stick to the skin, should be half an inch wide, and put on as warm as it can be borne. The patient should be got ready as much as possible before the poultice is brought to him, but he should remain covered with the bed clothes until the poultice is absolutely at the bedside. Always rub the skin dry with a warm towel when one poultice is removed and before applying the next. This prevents itching, and to a certain extent also that troublesome thing known as "poultice rash." The time for a large poultice to remain on is two to four hours; for a small one, one to two hours. A jacket poultice is one which is applied to the whole surface of the chest, front and back. It should be made in two halves, which can be united by strings at the corners, so that it is not necessary to uncover the front of the chest while applying the first half to the back. Mackintosh and jaconet outside a poultice make it retain its heat considerably longer. When poultices are discontinued cotton wool must be worn by the patient for some days after, but should be left off a day or so before he goes out of hospital. Ask, if you have any doubt about it, it is a point which the house surgeon, with his many duties, is apt to forget.

An ice-bag to the chest is frequently ordered. Here again, although it may seem paradoxical to say so, it is important to avoid chill. Because cold is to be applied to a certain part of the chest, it is not necessary to expose a larger portion of its surface than is required for the ice-bag. A blanket should cover the patient under the bedclothes, and touch the bag all round its circumference. An ice-bag is meant to apply cold; if, however, all the ice melts, not being at once replenished, the bag of water rapidly becomes warm, and acting like a poultice, does of course more harm than 'good. It takes a long time to remove the bad impression which a doctor gets of you, when he finds his cold applications transformed into hot ones.

Blisters are used chiefly in chronic affections. They are applied either with blistering plaster—emplastrum lyttæ—cut the size and shape required; or, blistering fluid—liquor epispasticus—is painted on. Briefly, the special points in applying blisters are as follow: Remove the natural grease of the skin by washing the part first with soap and warm water. When applied, cover the plaster or fluid with a little cotton wool retained in its place by a few strips of sticking plaster about ¼ in. wide. In using the fluid, in order to prevent it running, paint a circle of oil around the part to which it is to be applied, also a piece of wool in the direction in which it might run, should this accident happen in spite of your precautions. It looks bad to see a nurse's fingers covered with blisters, for having forgotten to place the wool in position, her first impulse is to stop it running with her fingers. A blister should rise in from five to six hours. Should it not do so, a small poultice applied over it will generally bring about a satisfactory result. To dress a blister, place a receiver, or piece of absorbent wool, underneath; snip the most dependent part with scissors, but do not cut cut off the cuticle; and when the fluid has all run out dress with boracic ointment. If the blister is ordered to be kept open, then you cut off the cuticle and dress it with the irritant ordered. This must be spread on lint cut exactly the size of the blister and no larger.

Leeches are much more used in some hospitals than others, and there are several details in applying them which it is necessary for you to know. The part to which they are to be applied is to be well washed first. The leeches are to be handled as little as possible. They are best placed in a wine glass which you then invert over the spot where you wish them to bite. If they refuse to bite, smear the surface over with milk, or sugar and milk, or cream, or try another leech. A nurse is not justified in pricking or scratching any patient's skin under any circumstances, as is often recommended, in order to make them bite. To ensure them biting at an exact spot, as behind the ear, half fill a test tube with cotton wool, then drop the leech tail first into it, and invert over the spot required. You may be frightened of a leech yourself—if so, don't let the patient notice it, as fright is catching—but familiarity, especially with a leech, breeds contempt. Nurses frequently have great difficulty in knowing which is the head end of the animal. The head is the more pointed end, but you may find out by letting him "walk." A leech always moves head first. When replete, they drop off of themselves, but should they be tardy in doing so, it is only necessary to sprinkle on them a little salt. Never pull them off or you will leave their teeth in the wound and also hurt your patient. After the leeches have dropped off it is often only necessary to apply a little absorbent wool to stop the bleeding; a very vigilant watch must, however, be kept on the punctures for some hours after, for they sometimes bleed profusely. Should they continue to bleed, there are several things you may do before sending for a doctor. It may be quite sufficient to wash the part clean with warm water, and expose it to the air. Other methods are simple pressure with the finger for a few minutes, either before or after stuffing the little holes with cotton wool, bringing the edges together with strapping, painting the punctures with collodion or tincture of perchloride of iron, and the application of ice. Supposing, however, all these have been tried and failed, you must send for a doctor. Probably he will touch it with caustic, the end of a hairpin heated to redness in a spirit lamp, or even pass a hare-lip pin under it.

I described to you how the apices of the lungs extended up above the collar-bone. In case you should be told to paint the one apex with iodine, how much should you paint? Supposing you were to stand behind the patient, and cover this part with your hand, so that the tips of the fingers just touched the collar-bone, the surface covered by your hand would be the right area to paint. It is generally ordered to be done every day, but you must be careful to ask whether it shall be continued, should the skin get very tender or red in the neighbourhood. The base of the lung is the part between the lower angle of the shoulder bone—scapula—and the last rib.

A linctus is a cough medicine of more or less syrupy consistency. Doctors often order a teaspoonful to be given "occasionally" when the cough is troublesome. As many of these medicines contain opium, an interval of an hour and a-half should be allowed to elapse between each dose, unless you have more definite orders.

Various substances are used as inhalations in chest diseases. An inhalation is a vapour of some drug or mixture of drugs. They are said to be used "moist" or "dry." Moist inhalations are those which are ordered to be added to boiling water and inhaled with steam two or three times a-day. An inhalation is said to be dry when, although it is a liquid, it is used with one of Dr. Burney Yeo's oronasal inhalers and inhaled continuously. The inhalers consist of little triangular boxes of perforated zinc, fitting over the patient's mouth and nose, containing a small piece of sponge, and fastening behind the patient's ears with elastic. The inhalation is poured about ten or twenty drops at a time on the sponge, and must be renewed as soon as it has evaporated, which will be about every twenty minutes or so. It is usual to give the patient the bottle and teach him to replenish it himself.

Powders may have to be ignited and the smoke inhaled. If there is great difficulty in igniting them, add a little saltpetre, when you will find that they burn merrily. Other substances, such as nitrite of amyl, are kept in glass capsules, which have to be broken when required. Full directions are, however, always to be found on the box. These last two sets of remedies, powders and capsules, are used chiefly in asthma. Let me again repeat that all remedies for asthma should be ready at a moment's notice.

Emetics are occasionally given in bronchitis, because the act of vomiting helps the expulsion of secretion from the chest. I just mention it here, so that you may understand the reason of it, should you see it done at any time.

### Everybody's Opinion.

*[Correspondence on all subjects is invited, but we cannot in any way be responsible for the opinions expressed by our correspondents. No communications can be entertained if the name and address of the correspondent is not given, or unless one side of the paper only be written on.]*

### THE HOSPITAL SHIP "HAMADRYAD," CARDIFF DOCKS.—A HINT TO LORD BUTE.

Miss Rose Blennerhasset, nurse superintendent of the Mission Hospital, Cardiff, writes as follows under date 22nd ult. : May I ask you to give a small space in your columns for the following? Cardiff is a rising town of about 130,000 inhabitants; it owes its wealth chiefly to its daily-increasing commerce, and the docks form the chief feature of the town. This being so, it seems reasonable to suppose that a fine hospital for seamen would be an absolute necessity. In point of fact, the only provision made for sick and disabled sailors is a hospital ship, a semi-rotten old vessel, and 50 to 60 patients are often crowded into a space only fit to accommodate 30 to 35. The ship is embedded in mud; it floats at high-water (spring-tides). On stormy days the motion has become violent enough to overturn furniture. The wards are low. A man above middle height cannot walk upright in them; they are not ventilated at all—the smell is simply dreadful, and an open window lets in equally bad smells, especially in summer, when the heat underneath the low, black roof is almost unbearable. The wards are disorderly and dirty; the patients and beds also look dirty and untidy. Two untrained men, who seem most kind and obliging, do all the nursing and cleaning, with what help can be got from the patients. "It is better than the fo'castle of a ship," said one of the persons in charge to me. Surely this is the "faint praise" that damns! The food looks most untempting—roughla cooked and served. No service is held in the ship; the English sailors see no clergyman. I believe the Norwegian and Swedish pastors visit the hospital, but the smells keep out most visitors. The doctor and his wife are, I hear, kindness itself, but powerless to effect any real change for the better. People assure me that Lord Bute intends taking the matter up, and that the town is waiting for his initiative. I do not know who is responsible for the existing state of things, but I am quite sure that it is a disgrace to Cardiff, and to all connected with it, and should be altered, and that quickly.

### WHO WILL HELP?

A medical superintendent writes: May I venture to ask the advice, and if possible the assistance, of some of your readers in the following case? One of our house porters, a thoroughly honest reliable fellow—has an idiot child five years old, his only boy, alas! The father is very anxious to get him into some home or asylum where he can be well cared for, and what little intelligence he has cultivated; but being only in receipt of 20s. a-week and his own rations, and having a wife and two other children to provide for, he is at a loss to know what to do. I have made enquiries, but the lowest charge seems to be about 15s. a week, which is, of course, impossible for him to pay. It should be mentioned that the child has lately become very restless, and is getting almost more than the mother, a poor weakly soul with two other children to do for, can manage. The anxiety and worry is praying on the father's mind, and prevents his discharging his duties with the precision for which he has hitherto been distinguished. Any advice or assistance would be most gratefully received by the Editor, The Lodge, Porchester Square, W.

### Examination Questions.

Over twenty answers were received to the February question, that of Miss Coborne being distinctly the best had she not forgotten the rule which says the answers must be short. As her paper was too much like a lengthy medical treatise, the prize must go to Nurse Annie Waind, of the Leicester Institute, to whom we have sent "Artificial Feeding and Foods," by Dr. W. B. Cheadle. So many answers were disqualified by the breaking of some rule, that we here repeat the rules for the guidance of future competitors—(1) Answers must be written on one side of the paper only. (2) They must not exceed 500 words. (3) They must must be addressed to the Editor of The Hospital, 140, Strand, London, W.C., the word "Nursing" being written on the envelope. (4) They must deal with the subject solely from the nursing point of view. (5) They must bear the full name and address of the sender. (6) Answers must not be copied from any book. The nurse who last month stated that salt sprinkled on a wound was very beneficial and that our grandmothers used cobwebs with great success had better have confined her display of knowledge to what she saw in hospital; there neither cobwebs nor salt are used to arrest hæmorrhage. Again, the nurse who wrote "vascular sedatives, such as aconite, should be administered" was treading too closely on the doctor's heels. Another nurse dealt with the subject solely as a midwife, and her writing was bad, and her punctuation conspicuous by its absence. Many of the answers were exceedingly good, especially one from Nottingham, and one from New Cross. For this month the question is: "Mention some of the divisions of fevers, explain the terms, and give examples." Answers must reach the Editor by March 12th. This month's prize answer will be printed next week.

### Presentation.

AT a farewell social evening given by Dr. and Mrs. Sweeting to the nursing staff of the Western Hospital, Fulham, on the eve of his departure to take up the duties of medical inspector of the Local Government Board (which appointment he has recently obtained), he was presented on behalf of the staff department by Surgeon-General Bostock, C.B., the chairman of the hospital, with a handsome silver coffee-pot. Dr. Sweeting acknowledged the gift on behalf of his wife and himself in a few appropriate words.

### Death in our Ranks.

THE death is announced of Sister Annette, of the Community of St. John the Baptist, Clewer. For over twenty years Sister Annette had acted as superior of the St. Raphael Convalescent Home at Worthing.

### New Appliances.

WE have received two ladies' abdominal belts from Madame Wood, of 285, Regent Street. The belts are neat and efficient, very light, weighing only 4 oz., and not expensive. The cheaper belt, Madame Wood says, she can supply to hospitals at 10s. each, and remembering how some twenty years ago these belts could never be obtained under three guineas, we think the sisters of obstetric wards should rejoice to hear this news.

### Notes and Queries.

TO CORRESPONDENTS.—1. Questions may be written on post-cards. 2. Advertisements in disguise are inadmissible. 3. In answering a query please quote the number. 4. If a private answer is desired, a stamped addressed envelope must be forwarded.

NOTICE.—We must really ask our correspondents to be more particular in enclosing name and address. Constantly we receive queries or letters with no name attached.

### Queries.

(55) *Nourishing Drinks.*—"Field" writes for suggestions for nourishing drinks for a patient with malignant tumour of the throat, who can take nothing but fluid.

(56) *Ambulance Classes.*—Are there ambulance classes held near Wandsworth?—*B. A. T.*

(57) *Charts.*—Can I get temperature charts cheaper than 1s. a dozen? —*Nurse Gerard.*

(58) *Rolling Bandages.*—Which is the best and simplest machine for rolling bandages?—*Mrs. B.*

(59) *Temporary Work.*—What employment can I get for twelve months while waiting to become a nurse?—*W. E. V.*

### Answers.

(50) *District Nursing.*—Sister Dora, Scarborough, is very pleased to give her experiences with respect to the best kind of boots and umbrellas for district work. If a county district nurse is suffering the discomfort Sister Dora did before she made the discovery of suitable boots, she sincerely pities her. The best and cheapest boots are those supplied by the India-rubber Boot Company (Dick's), who have shops in all large towns. The boots are only 6s. 6d. per pair, and are as good in appearance as ladies' shooting boots at a guinea a pair. Sister Dora has worn them for the last three years in district work, and has found them the greatest comfort; the soles are of rubber. There is another kind of boot, costing 10s. 6d., of which it is as well to have a pair for very wet weather—gutta-percha Wellingtons coming up to the knees, and so keeping the legs, and ankles dry. They are lined with flannel and have heels. With regard to the umbrella question, Sister Dora never carries one, finding it better to have a large hood to a cloak, which she wears over her head when it is wet weather, the cloak of waterproof cloth. It is not usual for boots to be supplied as uniform.

(56) *Ambulance Classes.*—Write to the secretary of the St. John's Ambulance Association, St. John's Gate, Clerkenwell, E.C. Classes begin to-day (February 28th) at the Church of England Young Men's Society, Leopold Rooms, 3, St. Bride Street, Ludgate Circus, E.C. Women's "First Aid" at half-past seven p.m.; nursing, eight p.m. Classes are also being held at Ravenscourt Gymnasium, by Dr. Forbes Winslow, and Hammersmith, Chiswick, and Fulham, are all arranging classes.

(57) *Charts.*—You can get them for 1s. for 50 from Messrs. Danielsson and Co., 52, Beaumont Street, Portland Place, London, W.

*D. D.* must send name and address.

*Mrs. Field.*—We do not supply nurses; you had better advertise in our pages.

### Vacancies.

THE following vacancies are announced:

### Matron.

Gorleston Cottage Hospital; £35. Western General Dispensary; £40.

### Night-Superintendent.

Queen's Hospital, Birmingham; £30. Bath Royal United Hospital; £75.

### Nurses.

Arbroath Poorhouse; £25.
Aimee Nursing Homes.
Bristol Infirmary Institute.
Burton-on-Trent Institution; £25.
Brixton Institute.
Burton-on-Trent Infirmary; £25.
Bournemouth Nursing Institute; £25.
Bedfordshire Trained Nurses' Institute; £28.
Bristol District Nurses' Society.
Croydon Institute.
Central London Throat Hospital.
Chester Deaconess House; £24.
Central Throat and Ear Hospital, W.C.; £20.
General Nursing Institute.
Greenwich Union Infirmary; £20.
Hillhead Trained Nurses' Home, Glasgow; £30.
Jersey Institute; £25.
Kent Nursing Institution; £30.
Kent and Canterbury Hospital; £25
Liverpool Eye Infirmary; £20.
Lincoln County Hospital; £20.
Middlesbrough Fever Hospital.
Manchester Sick Poor Institute; £25.
Mr. Wilson's Institution.
Mold Cottage Hospital; £25.
Nurses' Home, Hull.
Nursing Institute, Newport, I.W.
North-West London Hospital; £24.
Newark Hospital; £18.
Paddington Green Hospital.
Pendlebury Hospital; £20.
Ryde Infirmary; £18.
St. Leonard's Nurses' Home.
Scarborough Institute; £25.
Swansea Institute; £25.
St. George's Home, Sheffield.
South Hants Infirmary; £13.
St. Saviour's Infirmary, S.E.; £20.
South Metropolitan School District; £25.
Tewkesbury Hospital.
Victoria Park Hospital; £20.
Victoria Home, Bournemouth; £25.
Wimbledon Convalescent Hospital; £22.
Workhouse Infirmary Association.
Weston-Super-Mare Nursing Institute.
West Bromwich District Hospital, £22.

### District Nurses.

Burton-on-Trent Nursing Institution; £25.
Chelsea and Pimlico Nursing Associations.
Great Bedwyn; £25.
Penmaenmawr, North Wales.
South London Association.
St. George's Home, Sheffield.

### Probationers.

Bristol Royal Infirmary.
add dis nurses
Batley Cottage Hospital.
Burton-on-Trent Institution.
Halifax Infirmary.
Haverfordwest Infirmary.
Kent Nursing Institution.
Newark Hospital.
North-West London Hospital.
Nottingham Hospital for Women.
Samaritan Hospital for Women, Nottingham.
Salisbury Infirmary.
Southport Infirmary.
Weston-Super-Mare Hospital.

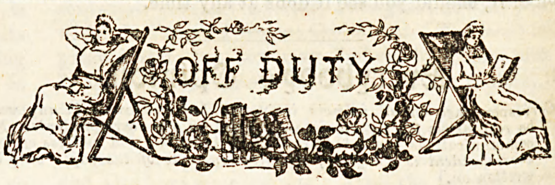

### Amusements and Relaxation.

### FOURTH QUARTERLY WORD COMPETITION

**Commenced Jan. 4th, 1890, ends March 29th, 1890.**

Three prizes of 15s., 10s., 5s., will be given for the largest number of words derived from the words set for dissection.

Proper names, abbreviations, foreign words, words of less than four letters, and repetitions are barred; plurals, and past and present participles of verbs, are allowed. Nuttall's *Standard* dictionary only to be used.

N.B.—Word dissections must be sent in WEEKLY, arranged alphabetically, with correct total affixed.

The word for dissection for this, the NINTH week of the quarter, being

"BLACKBIRDS."

| Names. | Feb. 20. | Totals. | Names. | Feb. 20. | Totals. |
|---|---|---|---|---|---|
| Holland | 25 | 643 | Nurse Clague | — | 40 |
| Red Cape | — | 132 | Lauriston | 23 | 643 |
| Nurse Marie | 18 | 446 | B. E. T. | 15 | 453 |
| Garfield | | 243 | Jacko | — | 110 |
| Midget | 23 | 602 | Lady Clara | — | — |
| E. M. S. | — | 149 | Patience | 24 | 675 |
| Sister | — | — | Ecila | 22 | 665 |
| Duchess | 24 | 437 | Maude | 23 | 628 |
| Ellice | 22 | 645 | Esperance | 25 | 653 |
| Reldas | 24 | 668 | Ruby | 20 | 634 |
| Jenny Wren | 25 | 590 | M. W. | 25 | 653 |
| Mopus | — | 122 | Crystal Palace | — | — |
| Neptune | — | 118 | Jackanapes | 19 | 507 |
| Qu'appelle | 24 | 634 | M. M. M. | — | 25 |
| Whitehoe | 21 | 97 | A. F. | 20 | 318 |

### Notice to Correspondents.

N.B.—All letters referring to this page which do not arrive at 140, **Strand, London, W.C.,** by the *first post on Thursdays*, and are not addressed PRIZE EDITOR, will in future be disqualified and disregarded.

